# Dual Coronary Artery Thrombosis: An Unforeseen Angiographic Encounter

**DOI:** 10.7759/cureus.66730

**Published:** 2024-08-12

**Authors:** Ammar Farook, Mohammed S Abdelghani, Omar S Makawi, Ihsan Rafie

**Affiliations:** 1 Cardiology, Hamad Medical Corporation, Doha, QAT

**Keywords:** cardiogenic shock, multivessel coronary artery disease (mvcad), primary percutaneous intervention, dual coronary artery infarction, st-elevation myocardial infarction (stemi)

## Abstract

Acute myocardial infarction (AMI) frequently involves single-vessel coronary artery disease, but simultaneous thrombosis in multiple coronary arteries is a rare and challenging clinical scenario. We report the case of a 42-year-old Southeast Asian male with a six-month history of hypertension controlled by a single antihypertensive agent, presenting to the emergency department with central chest pain radiating to the back. The initial electrocardiography (ECG) showed ST elevation in the inferior leads. Primary percutaneous coronary intervention (PCI) via the right femoral approach revealed complete thrombotic occlusions in the left anterior descending (LAD) and right coronary artery (RCA). Drug-eluting stents (DES) were deployed, restoring thrombolysis in myocardial infarction (TIMI) III flow. Despite initial hemodynamic stability, the patient experienced cardiogenic shock (CS), necessitating a relook angiogram that confirmed patent stents and identified an additional stenosis in the first diagonal branch (D1). An intra-aortic balloon pump (IABP) was inserted. The patient’s course was complicated by recurrent CS, septic shock secondary to *Fusobacterium periodonticum* bacteremia, acute kidney injury, multiple supraventricular arrhythmias (SVTs), and partial thrombosis of the right radial artery leading to dry gangrene of the right index and thumb fingers. He was eventually discharged on oral warfarin for radial artery thrombosis and paroxysmal atrial fibrillation with follow-up care with vascular surgery.

## Introduction

Patients with acute myocardial infarction (AMI) often exhibit multivessel coronary artery disease on coronary angiography (CAG). Plaque rupture can subsequently lead to ST-elevation myocardial infarction (STEMI), and multiple plaque ruptures have been documented in autopsy reports [1]. However, the occurrence of multiple acute thrombus formations in patients presenting with AMI is relatively rare, occurring in approximately 2.5% of all primary percutaneous coronary intervention (PCI) patients [2]. These patients frequently exhibit hemodynamic instability, with 28% presenting in cardiogenic shock (CS), 22% experiencing life-threatening ventricular arrhythmias (VTs), and 22% requiring intra-aortic balloon pumps (IABPs) [2]. The low incidence is partly attributed to many patients succumbing to sudden cardiac death (SCD) before hospital admission, with autopsies of SCD patients revealing multiple thrombi in up to 50% of cases [3]. Additionally, these patients often face complicated post-intervention hospital courses [4]. Recognizing and managing such cases promptly is crucial to improve outcomes. We present a case of dual coronary artery thrombosis that resulted in a challenging intervention and necessitated a relook angiogram due to lack of anticipated clinical recovery.

## Case presentation

A 42-year-old gentleman, a nonsmoker and nonalcohol drinker from Southeast Asia, with a six-month history of hypertension controlled by a single antihypertensive agent, presented to the emergency department complaining of central chest pain radiating to the back, which began three hours prior. He had a family history of coronary artery disease in his father, although the details are unknown. Aside from an allergy to ibuprofen, the rest of his medical history was uneventful.

**Figure 1:**
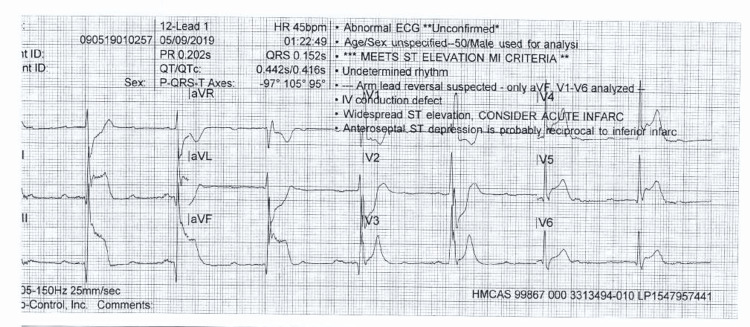
Twelve-lead electrocardiography (ECG) on arrival to the emergency department showing ST elevation in leads II, III, AVF, V4-V6, with ST depression in leads I, AVL, V1, and V2 AVF: augmented vector foot; AVL: augmented vector left

According to first responders, the patient was found lying supine on his couch with severe back pain and multiple episodes of vomiting. The circumstances prior to the onset of chest pain are unknown, but this was the first such episode he had ever experienced. In the ambulance, the initial electrocardiography (ECG) (Figure 1) showed ST elevation in the inferior leads, and the patient received intravenous (IV) fentanyl and a single dose of atropine for symptomatic bradycardia, after which his heart rate improved. Due to intractable vomiting, he did not receive loading doses of aspirin or clopidogrel.

**Figure 2:**
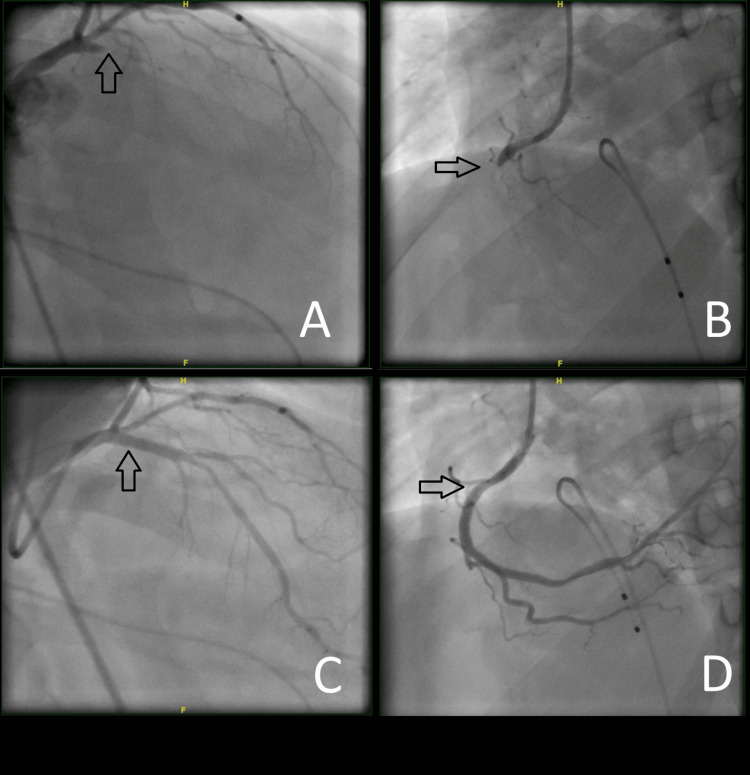
(A) Thrombus formation in the ostial left anterior descending artery (LAD) in right anterior oblique view. (B) Thrombus formation in the proximal right coronary artery (RCA) in the left anterior oblique view. (C) Percutaneous coronary intervention (PCI) to the LAD. (D) PCI to the RCA

Upon arrival at our emergency department, he was lethargic yet hemodynamically stable. A repeat ECG confirmed ST elevation in the inferior (II, III, augmented vector foot (aVF)) and lateral leads (I, augmented vector left (aVL)), along with reciprocal changes in leads V2-V3 and ST elevation in right-sided, posterior leads. Subsequently, the patient became bradycardic, hypotensive, and desaturated, leading to intubation and primary PCI via the right femoral approach. Angiography revealed a right-dominant circulation and two complete thrombotic occlusions in the left anterior descending artery (LAD) and right coronary artery (RCA), respectively, with thrombolysis in myocardial infarction (TIMI) 0 flow. Predilation was performed using a Maverick 3.0 X 12 mm compliant balloon in both lesions. Subsequently, a 4 X 18 mm (Xience Sierra) drug-eluting stent (DES) was deployed in the proximal RCA, while another 3.5 X 15 mm DES (Resolute Onyx) was inserted in the proximal LAD (Figure 2A-2B). Both lesions appeared to be acute, spontaneously developed thrombi. The final result was TIMI III flow across both coronaries (Figure 2C-2D). The patient was found to be in complete heart block, necessitating the insertion of a temporary pacemaker wire in the Cath Lab.

**Figure 3:**
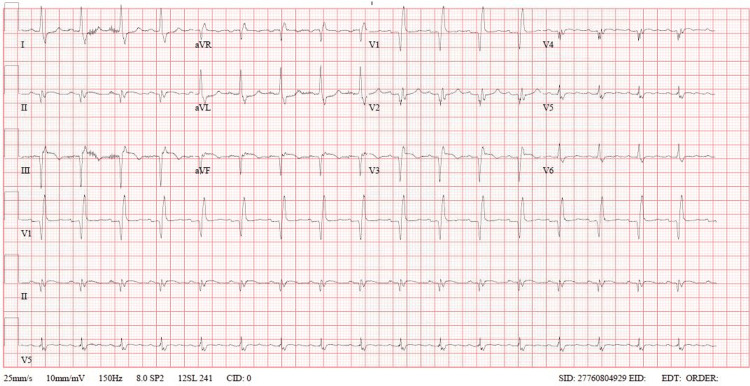
ECG Showing sinus tachycardia with ST elevation in lead III, AVF, and V3 with reciprocal changes in lead I and AVL AVF: augmented vector foot; AVL: augmented vector left

The patient was admitted to the coronary intensive care unit (ICU), intubated on mechanical ventilation, and sedated after being started on eptifibatide infusion. However, he soon developed profuse epistaxis, mucosal bleeding, and blood-tinged urine, leading to the discontinuation of the infusion. Initial investigations revealed mild leukocytosis and a high-sensitivity troponin T peak of 77,000. The patient had a normal HbA1c and lipid profile. A post-PCI ECG done a few hours later (Figure 3) showed evolving ST-T wave changes with sinus tachycardia.

**Figure 4:**
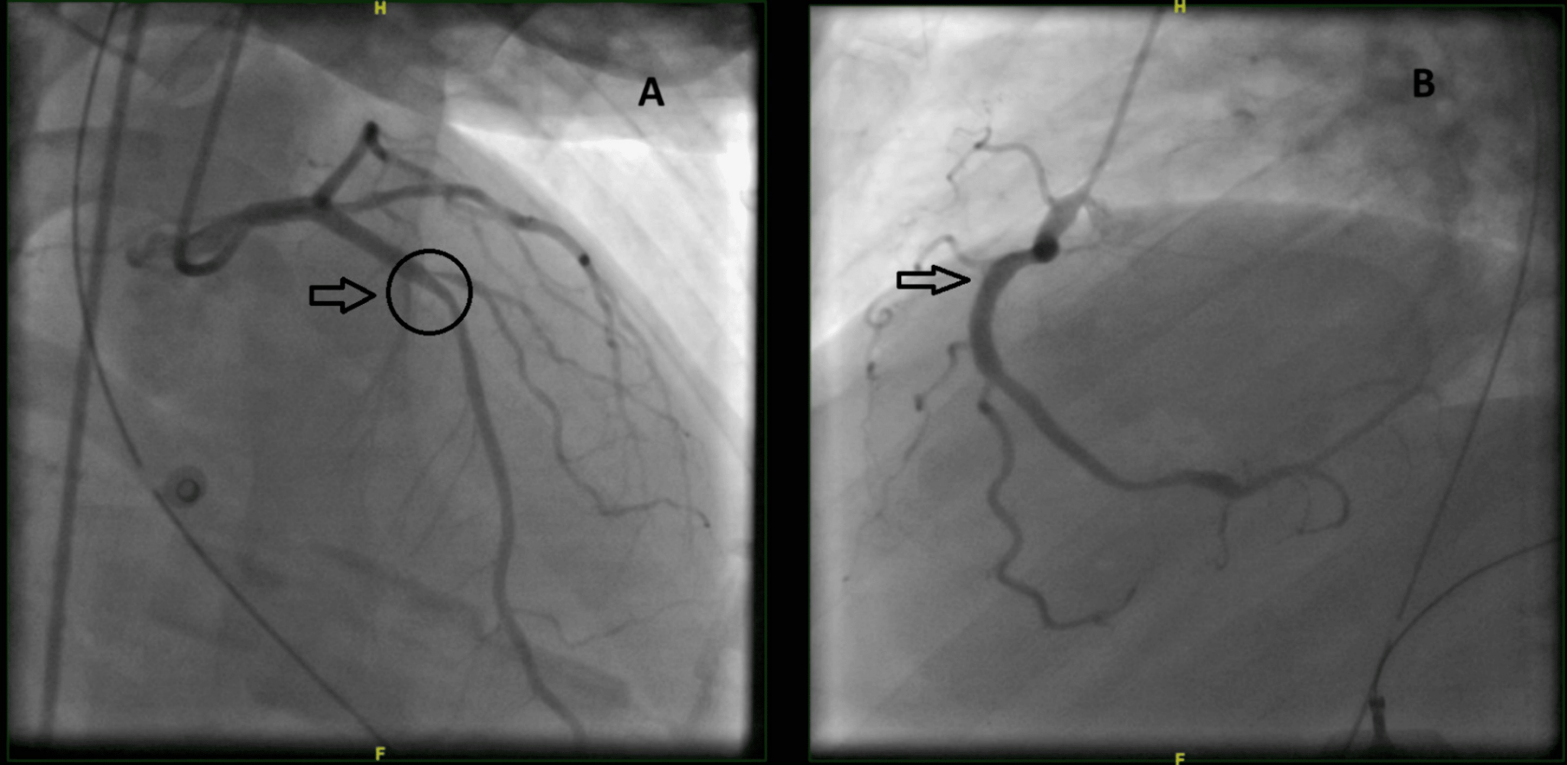
(A) Patent LAD stent with D1 stenosis. (B) Patent RCA stent LAD: Left anterior descending artery; RCA: right coronary artery

An initial echocardiogram demonstrated an ejection fraction (EF) of 33%, grade II diastolic dysfunction, severely reduced right ventricle (RV) function, and akinesia of the apical and inferior segments. The patient was not actively bleeding and subsequently received loading doses of aspirin and clopidogrel. Soon after, he became hypotensive, did not respond to fluid challenges, and did not improve on three vasopressors and inotropes. Therefore, on the same day, he was taken for a relook coronary angiogram to rule out the possibility of acute stent thrombosis due to the delay in the initiation of antiplatelet agents. The angiogram revealed patent stents in the LAD and RCA, with an additional 60% stenosis in the first diagonal branch (D1) (Figure 4). An IABP was inserted.

The remainder of his hospital course was significant for another episode of CS after the removal of the IABP, septic shock treated with a course of IV antibiotics for *Fusobacterium periodonticum* bacteremia, and acute kidney injury (AKI). He also developed multiple episodes of atrial fibrillation with a fast ventricular rhythm, supraventricular tachycardias (SVT), and intermittent atrial flutter during his subsequent course post-extubation. Finally, the patient developed partial thrombosis of the right radial artery, leading to dry gangrene of his right index and thumb fingers. He was eventually discharged on oral warfarin for his radial artery thrombosis and paroxysmal atrial fibrillation, with follow-up with vascular surgery.

## Discussion

STEMI occurs when a vulnerable atheromatous plaque ruptures, leading to partial or complete thrombosis of a coronary artery, known as the "culprit" vessel. However, acute simultaneous thrombosis in multiple coronary arteries (leading to multiple culprits) in patients presenting for primary PCI is a rare angiographic finding, occurring in approximately 2.5% of cases [2]. Many coronary plaques may be destabilized simultaneously by an inflammatory response that does not target a specific lesion. Because atherosclerosis is a chronic inflammatory disease that frequently manifests as an acute coronary syndrome (ACS) and can be multifocal, rupturing numerous coronary plaques, the term pancoronaritis appropriately describes the condition. According to Goldstein et al., over 30% of patients with STEMI had multiple plaque ruptures with overlying thrombi [5].

Identifying at-risk patients prior to CAG is challenging, as common risk factors for multiple culprit lesions include male gender and tobacco usage [2]. Additional factors such as hypercoagulable states, cocaine use, essential thrombocytosis, oral contraceptive pills, tamoxifen, and clopidogrel resistance due to CYP2C19 polymorphism may contribute to the development of multivessel coronary artery thrombosis, though the exact cause often remains unknown [5]. Rarely, autoinflammatory conditions such as Behcet's disease can cause dual vessel acute thrombosis [6].

ECG findings may not always indicate the acute involvement of multiple coronary arteries. In a review of 47 cases with multiple culprit arteries, one-third had isolated inferior STEMI on initial ECG, while half were later found to have both RCA and LAD acute thrombosis on CAG, similar to our patient’s presentation [2]. The LAD and RCA are the most commonly implicated vessels in dual coronary artery thrombosis [2,3,4,7]. Compared to patients with single complex plaques, those with multiple complex plaques are at increased risk of recurrent ACSs, repeat PCI, particularly of non-infarct-related lesions, and coronary artery bypass grafting [8].

Although modern PCI techniques have reduced the fatality associated with multiple acute coronary thrombi [2], early identification of patients with dual coronary artery thrombosis remains crucial due to the high rates of periprocedural complications such as CS and arrhythmias. Our patient experienced CS, necessitating a relook angiogram to rule out in-stent thrombosis, insertion of an IABP, management of SVTs, and secondary adverse outcomes from multiple angiograms such as radial site thrombosis and AKI. CS is common in these patients, with reported frequencies of 36% [2] to 41% [9] at presentation. VTs are more frequently encountered than supraventricular ones [7]. Furthermore, approximately one-third of these cases required IABP insertion [2,9], underscoring the importance of early anticipation and intensive care observation in these patients.

## Conclusions

Dual coronary artery thrombosis, once discovered on coronary angiogram, poses significant challenges as patients are at risk of complications such as CS and an increased need for repeat coronary angiograms due to lack of clinical improvement. This can lead to additional risks of adverse events, including AKI. Given that ECG at presentation may misrepresent the presence of multiple culprit lesions, further studies are needed to discover potential ways to anticipate this finding for improved patient outcomes.
